# Thanks to Editorial Board of Arquivos de Neuro-Psiquiatria

**DOI:** 10.1590/0004-282X-ANP-2022-A001

**Published:** 2022-02-28

The Editors of Arquivos de Neuro-Psiquiatria thanks to **Associate Editors** (70), **Editorial Board** (52) and **Referees** (506) who altruistically and without payment have expended their time to develop the editorial tasks of this journal since January 1st , 2021 to December 31st , 2021.

Thank you very much!

## ASSOCIATE EDITORS

Ayrton Roberto Massaro

Adriana Bastos Conforto

Adriana Moro

Alexandra Prufer Queiroz Campos Araújo

Ana Carolina Coan

Analuiza Camozzato

Antonio José da Rocha

Antonio Lucio Teixeira

Carlos Henrique Ferreira Camargo

Carlos Otto Heise

Carolina de Medeiros Rimkus

Celi dos Santos Andrade

Chien Hsin Fen

Clarissa Lin Yasuda

Cristiane Nascimento Soares

Dalva Lucia Rollemberg Poyares

Daniel Ciampi de Andrade

Edmar Zanoteli

Eduardo Genaro Mutarelli

Elisa de Paula França Resende

Ethel Mizrahy Cuperschmid

Fábio A. Nascimento

Fabíola Dach

Fernando Spina Tensini

Francisco de Assis Aquino Gondim

Gabriel Rodriguez de Freitas

Grace Schenatto Pereira Moraes

Iscia Teresinha Lopes Cendes

Jamary Oliveira Filho

José Luiz Pedroso

Juliana Gurgel-Giannetti

Karen Nunez-Wallace

Karina Braga Gomes

Laura Silveira Moriyama

Lea Tenenholz Grinberg

Leandro Tavares Lucato

Lécio Figueira Pinto

Leonardo Cruz de Souza

Luciano De Paola

Luciene Covolan

Luís Otávio Sales Ferreira Caboclo

Marcondes Cavalcante França Jr

Marcos Christiano Lange

Maria Fernanda Mendes

Mario Fernando Prieto Peres

Marzia Puccioni-Sohler

Michael Hornberger

Mônica Sanchesz Yassuda

Octávio Marques Pontes Neto

Orlando Povoas Barsottini

Paulo José Lorenzoni

Paulo Pereira Christo

Pedro Andre Kowacs

Pedro Sampaio

Péricles Maranhão-Filho

Renato Puppi Munhoz

Rosana Cardoso Alves

Rosana Hermínia Scola

Sarah Teixeira Camargos

Sérgio Monteiro de Almeida

Sérgio Rosemberg

Sheila Cristina Ouriques Martins

Sônia Maria Dozzi Brucki

Suzana Maria Fleury Malheiros

Tarso Adoni

Vitor Tumas

Vivaldo Moura Neto

Wilson Marques Jr.

Yara Dadalti Fragoso

Ylmar Correa Neto

## EDITORIAL ADVISORY BOARD

Acary de Souza Bulle Oliveira

Alberto J. Espay

Alexis Brice

Andrea Slachevsky

Américo Ceiki Sakamoto

Andrew J. Lees

Bruce L. Miller

Bruce Ovbiagele

Carlos Alberto Mantovani Guerreiro

Carlos Roberto de Mello Rieder

Christina Marra

Didier Leys

Fernando Cendes

Fernando Kok

Francisco Cardoso

Giancarlo Comi

Gilmar Fernandes do Prado

Henrique Ballalai Ferraz

Hugh J. Willison

Jaderson Costa da Costa

João José Freitas de Carvalho

Joaquim Ferreira

Joaquim Pereira Brasil Neto

José Manuel Ferro

Lineu César Werneck

Luiz Henrique Martins Castro

Marcelo Eduardo Bigal

Márcia Lorena Fagundes Chaves

Marco Aurélio Lana-Peixoto

Marcos Raimundo Gomes de Freitas

Maria José Sá

Maria Lúcia Brito Ferreira

Marilisa Mantovani Guerreiro

Maurice Borges Vincent

Mônica Levy Andersen

Oscar Del Brutto

Oscar Gershanik

Osvaldo José Moreira do Nascimento

Osvaldo Massaiti Takayanagui

Pedro Chaná-Cuevas

Raimundo Pereira da Silva Neto

Regina Maria Papais-Alvarenga

Ricardo Allegri

Ricardo Nitrini

Roger Walz

Rubens José Gagliardi

Sérgio Teixeira Ferreira

Stefan Schwab

Ugo Nocentini

Umbertina Conti Reed

Vladimir Hachinski

Walter A. Rocca

## REVIEWERS

Abayuba Perna

Abdulkadir Tunç

Abelardo Queiroz Campos Araujo

Acary Souza Bulle Oliveira

Adriana Andrade

Adroaldo Rosseti

Ahmet Sevki Taskiran

Ailton de Souza Melo

Alaise Siqueira

Alberto Rolim Muro Martinez

Aleksander Erga

Alessandra Bastone

Alessandro Finkelsztejn

Alessia Pellerino

Alex Meira

Alex Silva

Alexandra Prufer de Queiroz Campos Araújo

Alexandre Maulaz

Ali Zeynel Abidin Tak

Alice Oliveira

Aline Ayres

Aline Carmo

Aline Chacon Pereira

Aline Miranda

Aline Mizuka Kozoroski Kanashiro

Aline Mourao

Alireza Zandifar

Alvaro Pentagna

Ana Beatriz Ayroza Galvão Ribeiro Gomes

Ana Borja

Ana Caline Nobrega

Ana Carolina Coan

Ana Carolina Zetehaku

Ana Lucila Moreira

Ana Paula Fontana

Ana Paula Gonçalves

Ana Paula Hamad

Anderson Paiva

André Felício

André Palmini

André Silva

Andreia Gomes Bezerra

Andreia Mendonça

Angela Dalbem

Anna Carolyna L Gianlorenço

Anna Pinto

Antonio Alberto Lopes

Antonio José da Rocha

Antonio Marrone

Antônio Zuardi

Antonio-Pedro Vargas

Aristeidis H. Katsanos

Artur Schumacher Schuh

Asuman Celikbilek

Ayrton Roberto Massaro

Berenice Silva

Bernardo Cacho-Díaz

Bilgin Öztürk

Blener Mateus

Boris Kotchoubey

Breno Barbosa

Bruna Dutra

Bruno Pedreira

Bruno Santos-Lobato

Caio Vinicius Simioni

Camila Corrêa

Camila Pupe

Caner Demir

Carina Tellaroli Spedo

Carla Andrea Caldas

Carlos Alberto Bordini

Carlos Bernardo Tauil

Carlos Brandão

Carlos Clayton Macedo

Carlos Eduardo Soares Silvado

Carlos Henrique Camargo

Carlos Mauricio Almeida

Carlos Monteiro

Carlos Otto Heise

Carlos Roberto de Mello Rieder

Carlos Senne

Carolina Candeias

Carolina de Medeiros Rimkus

Carolina de Oliveira Souza

Carolina Delgado

Carolina Rouanet

Carolina Souza

Caroline Folchini

Cengiz Özdemir

Cesar Lopes

Charles André

Chin An Lin

Chris Traner

Christina Marra

Clara Camelo

Clarice Tanaka

Claudia Altamura

Claudia Ferreira da Rosa Sobreira

Cláudia Maia Memória

Cláudia Vasconcelos

claudio queiroz

Cleonísio Rodrigues

Conrado Borges

Conrado Estol

Cristiana Borges Pereira

Cristiane Nascimento Soares

Cristina Iwabe-Marchese

Daiane Boff

Daniel de Castro Medeiros

Daniel Giansante Abud

Daniel Pimentel

Daniel Rubio de Souza

Daniela Godoi Jacomassi

Daniele Bernardes

Danielle Andrade

Danyella Dogini

Debora Bevilaqua Grossi

Débora Palma Maia

Deborah Cristina Gonçalves Luiz Fernani

Denis Bernardi Bichuetti

Denise Nicaretta

Deniz Sahin

Dharah Puck Bispo

Dieggo Jefferson Silva Melo

Diego Fragoso

Diogo Santos

Douglas E. Ney

Dr. Azizuddin Khan

Eberval Gadelha Figueiredo

Edmar Zanoteli

Eduardo Cavalcanti

Eduardo De Sousa

Eduardo Faveret

Eduardo Nicoliche

Eduardo Nogueira

Elcio Juliato Piovesan

Elen Beatriz Pinto

Eliana da Cunha

Eliasz Engelhardt

Elisa Resende

Elizabeth Comini-Frota

Elizabeth Quagliato

Emanuelle Silva

Emrah Dirican

Eric Pasmant

Erica Vieira

Esther Cubo

Ethel Ciampi

Ethel Cuperschmid

Euldes Mendes

Eva Rocha

Fabrizio Anniballi

Fabio Barroso

Fabio Fernandes

Fabíola Dach

Fabiola Lys Medeiros

Fabrício de Borba

Fabrício Diniz de Lima

Fazilet Ozdenoğlu

Felipe Aidar

Felipe Dantas

Felipe Oliveira

Felipe Pacheco

Felippe Borlot

Ferhat Balgetir

Fernanda Carvalho

Fernanda Teresa Lima

Fernando Figueira

Fernando Frassetto

Fernando Freitas

Fernando Kok

Fernando Morgadinho Coelho

Fernando Stelzer

Fidel Castro de Meira

Flavio Moura

Flávio Rezende Filho

Florindo Stella

Francisco de Assis Aquino Gondim

Francisco Dias

Francisco Eduardo Cardoso

Francisco José Montalverne

Francisco Luciano Pontes Junior

Francisco Sepulveda

Francisco Tellechea Rotta

Frederico Jorge

Frederico Moura

Gabriel Modolo

Gabriel Pires

Gabriel Rodriguez de Freitas

Gabriela Bandeira

Geraldo Rizzo

German Moris

Geruza Perlato Bella

Gilberto Alves

Gilberto Manzano

Gildo Santos Filho

Giovana Diaféria

Gisele Sampaio Silva

Gislaine Baroni

Giuseppe Minniti

Gonzalo Forno

Guilherme Mendonça

Guilherme Oliveira

Guilherme Sciascia do Olival

Guilherme Silva

Guilherme Silveira

Guilherme Valença

Guillermo Delgado-García

Gustavo Alarcão

Gustavo José Luvizutto

Gustavo Leite Franklin

Gustavo Moreira

Gutemberg Santos

Hansotto Reiber

Hazem Ashmawi

Hedley Knewjen Quintana

Hélcio Kanegusuku

Hélio A. G. Teive

Heloisa Alonso Matielo

Heloisa Viscaino Pereira

Henrique Ballalai Ferraz

Hércules Leite

Hidelberto Silva

Hideraldo Cabeça

Hugo Resende

Isabella Claudia Glitza Oliva

Isabella D'Andrea Meira

Iuri Neville

Ivan Aprahamian

Izabela Barbosa

Jacy Parmera

Jamary Oliveira Filho

Jamir Rissardo

Jan E. Kennedy

Janini Chen

Jaqueline Pereira

Jay Gavvala

Jefferson Becker

Jerusa Smid

João Amadera

João Brainer Clares Andrade

Joao Pinho

Jocemar Ilha

John Araujo

John Ebersole

John J. Halperin

Jonas Alex Saute

Jorge Burneo

José Antonio Fiorot Jr

José Carlos Galduróz

Jose Geraldo Speciali

José Garbino

José Luiz Pedroso

José Manuel Ferro

José Roberto Wajman

Joseane Bouzon

Jozelio Freire de Carvalho

Juliana Algemiro

Juliana Chichorro

Juliana Sallum

Juliana Tavares

Karina Braga Gomes

karmond Garson

Katia Lin

Katie Almondes

kenia campanholo

Kenzo Hokazono

Kette Dualibi Ramos Valente

Lara Oliveira

Larissa Salvador

Laura Guilhoto

Laura Silveira-Moriyama

Leandro Iuamoto

Leandro Valiengo

Lécio Figueira Pinto

Leonardo Carbonera

Leticia Fezer

Leticia Pereira de Brito Sampaio

Lígia Catai

Liliana Patrucco

Lina Velilla

Linamara Battistella

Lineu César Werneck

Lívia Almeida Dutra

Luana Antunes Maranha Gatto

Lucas Cabral

Lucas Mourão

Lucas Schilling

Luciana Cassimiro

Luciana Drummond

Luciano De Paola

Luciano Mariano

Luciano Simao

Luciene Covolan

Lucio Huebra

Luis Henrique Castro Afonso

Luiz Carlos Marrone

Luiz Felipe Rocha Vasconcellos

Luiz Paulo Queiroz

Magda Medieros

Magda Lohorgue Nunes

Maira Barbosa

Mamede de Carvalho

Manoel Sobreira-Neto

Manuel Jibaja

Manuelina Brito

Marcel Simis

Marcela Silagi

Marcelo Ares

Marcelo Cruzeiro

Marcelo Daudt von der Heyde

Marcelo de Brito

Marcelo Rugiero

Marcia Hallinan

Marcia Jardim

Márcia Lorena Fagundes Chaves

Márcia Silva

Marcio A. P. Pereira

Márcio de Oliveira

Marcio Moacyr Vasconcelos

Marcio Nattan

Marco Antonio Lima

Marco Aurélio Lana-Peixoto

Marco Gonzalez-Castellón

Marco Utiumi

Marcondes Cavalcante França Jr

Marcos Christiano Lange

Marcos Gomes de Freitas

Marcos Santos

Marcos Teles Gomes

Marcus Della Coletta

Marcus Gomez

Marcus Goncalves

Marcus Vinícius Pinto

María Carolina Sepúlveda Soto

Maria da Graça Martin

Maria Elisa Piemonte

Maria Emilia Andraus

Maria Isabel Amaral

Maria Manreza

Maria Montenegro

Maria Paula Foss

Mariana Artilheiro

Mariana Callil Voos

Mariana Cunha

Mariana Spitz

Marilisa Guerreiro

Marina Alvim

Marina C. Vinaud

Marina Farah

Marino Muxfeldt Bianchin

Mario Campero

Mario Cecchini

Mario Cornejo-Olivas

Mário Fernando Prieto Peres

Mário Luiz Monteiro

Marlise Ribeiro

Marlos Martins

Marta Rubiera

Marzia Puccioni-Sohler

Mateus Boaventura

Mateus Mistieri Simabukuro

Mateus Pellegrino

Maurice Mars

Mauricio Calomeni

Mauricio Franco Farez

Mauro Eduardo Jurno

Mauro Lúcio Leitão Condé

Mayara Machado

Mayela Rodríguez-Violante

melek ulusoy

Michael Funke

Michael S. Zandi

Michel Frudit

Millene Camilo

Mônica Parolin

Muhammed Akhlaq

Nasser Allam

Natalia Fiorenza

Nathália Bergamasco

Nayanne Bosaipo

Neslihan Altuntaş

Nevin Kuloğlu Pazarcı

Nilton Custodio

Nise Alessandra Sousa

Niures Matioli

Noe Alvarado-Vasquez

Norberto Frota

Octavio Marques Pontes Neto

Olagide Castro

Olivier Walusinski

Orlando Graziani Povoas Barsottini

Osvaldo J. M. Nascimento

Otto Fustes

Pablo Brea Winckler

Patricia Lillo

Paula Herrera

Paulo Eduardo Mestrinelli Carrilho

Paulo José Lorenzoni

Paulo Mei

Paulo Pereira Christo

Paulo Victor Sgobbi de Souza

Pedro André Kowacs

Pedro Augusto Sampaio Rocha Filho

Pedro Barbosa

Pedro Braga-Neto

Pedro Brandão

Pedro Castro

Pedro Cougo

Pedro Hamamoto Filho

Pedro Kurtz

Pedro Paulo Oliveira Carneiro

Pedro Ribeiro

Pedro Tomaselli

Péricles Maranhão Filho

Pericles Otani

Philip Glass

Rafael Carneiro

Rafael da Costa Monsanto

Raffaele Ornello

Raphael Cirino

Raphael Spera

Regina Alvarenga

Renan Barros Domingues

Renan Domingues

Renata Ducci

Renata Kochhann

Renato Luiz Marchetti

Renato Nickel

Renato Puppi Munhoz

Ricardo Nitrini

Ricardo Silva Pinho

Ricardo Weinlich

Rihab Borji

Roberta Roiz

Roberto Prado

Rodrigo Bazan

Rodrigo Rivera

Rogerio de Oliveira

Rogério de Rizo Morales

Rogério Oliveira

Rosana Hermínia Scola

Rubens Gisbert Cury

Rubens José Gagliardi

Rui Kleber Martins

Rui Reis

Ruy R. R. Campos

Samir Hanna

Sarah Teixeira Camargos

Sebastián Ameriso

Seema Jain

Seidel Lopez Guerra

Sérgio Antoniuk

Sergio Monteiro de Almeida

Serhat Tokgoz

Sheila Cristina Ouriques Martins

Sidney Gomes

Silke Anna Theresa Weber

Silvia Tenembaum

Simone Capellini

Simone Karuta

Sinem Sökücü

Soniza Vieira Alves-Leon

Stefan Schwab

Stephan Schuele

Suely Ferraciolli

Sule Aydin Turkoglu

Sumita Halder

Sylvia Ciasca

Tamine Capato

Tarso Adoni

Taysa Ribeiro

Thais Machado

Thaiza Agostini Córdoba de Lima

Thiago Cardoso Vale

Thiago Cerqueira Silva

Thiago William Jorge

Thomas Hoffmann

Tufan Mert

Valéria Passos

Valeria Scavasine

Vasileios Kokkinos

Vera Braatz

Veralice de Bruin

Victor Carlos Montalvan Ayala

Victor Hugo Marussi

Victor Rivera

Vinicius Guirado

Vinicius Montanaro

Vinicius Schoeps

Vitor Paes

Vítor Tumas

Viviane de Hiroki Flumignan Zétola

Wagner Mauad Avelar

Walter Santos Moraes

Wesley T. Kerr

William R. Tebar

Wilson Marques Jr

Wladimir Bocca Vieira de Rezende Pinto

Wyllians Borelli

Yara Dadalti Fragoso

Yessika Rojas

Ylmar Correa-Neto

Zalina Zahari

Zaza Katsarava

Zulfi Haneef

